# Description of an online hospital platform, China

**DOI:** 10.2471/BLT.18.226936

**Published:** 2019-06-03

**Authors:** Dan Wu, Therese Hesketh, Haihua Shu, Wannian Lian, Weiming Tang, Junzhang Tian

**Affiliations:** a Department of Clinical Research, London School of Hygiene & Tropical Medicine, London, England.; b Institute for Global Health, University College London, London, England.; c Guangdong Second Provincial General Hospital, 466 Xingang Middle Road, Guangzhou, China.; d The University of North Carolina at Chapel Hill Project-China, Guangzhou, China.

China has a three-tier health-care system; primary health-care facilities are expected to provide affordable first-contact care, while secondary and tertiary care facilities provide specialist referral services. However, with no gatekeeping in the primary health-care system, patients can freely choose their provider at any health facility, and many routinely use hospital outpatient services for first-contact care. Primary health care services in China face several challenges, including overprescribing of profitable drugs and diagnostic tests, competition for patients where there is fee-for-service^1^^,^^2^ and increasing demand for healthcare, especially in the context of an ageing population.

The Chinese government views digital health as a solution to address these challenges. A rapid increase in internet users ­ from 22.7% of the population in 2008 to 59.6% in 2018^3^ ­ provides an opportunity to develop online triage and consultation services. Here, we describe an online hospital platform in Guangdong province, the challenges the platform faces and the potential role that digital health can play in primary care.

The Guangdong Second Provincial General Hospital is leading the platform, referred to in China as an “internet hospital”. This platform was founded in 2012 and has been accredited by the Health Commission of Guangdong Province as the first internet hospital in China. The platform includes 700 licensed or assistant physicians from 19 county-level hospitals and the Guangdong Second Provincial General Hospital. These physicians have been re-trained and accredited through a general practitioner training programme. The physicians provide online video consultations for about 14 000 community-based health providers working in health centres, village clinics, university health services and pharmacies in Guangdong. Only patients who use these community-based providers have access to the platform. In 2018, an average of 33 000 visits per day were made to these connected community-based providers. The providers are motivated to use the platform because they are thus able to facilitate their patients’ access to health professionals and technology in higher-level hospitals. 

**Fig. 1:**
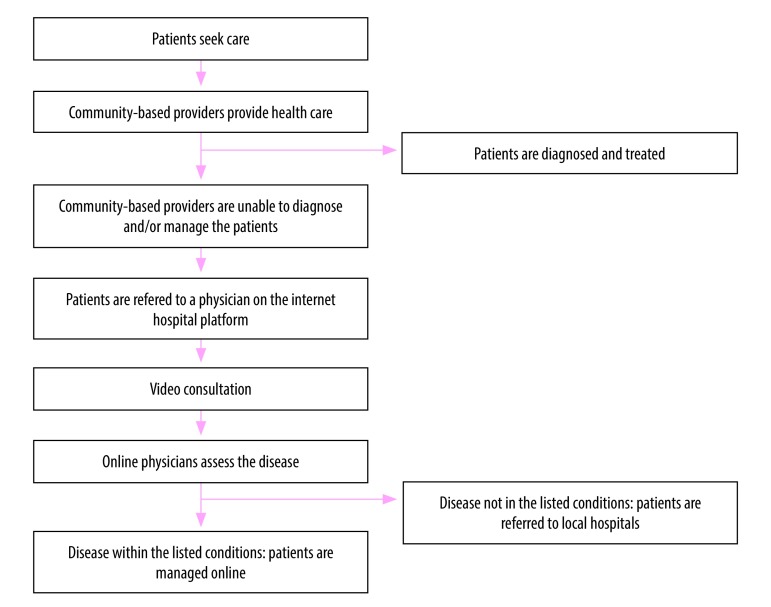
Medical consultation process for an internet hospital, China

The platform supports the primary health-care provider at the community level in diagnosis and treatment decisions. The provider can link the patient directly to the platform for a video consultation with a physician. If no diagnosis can be made, the patient is referred to a hospital associated with the platform (Fig. 1). The platform is expected to strengthen primary care and create a local gatekeeping mechanism. Inspired by the Guangdong experience, the Chinese State Council issued the first national directive on developing digital medicine in April 2018.^4^ The directive encourages hospitals to provide primary care and a referral system for patients with common conditions and chronic diseases through online platforms. This approach also acts as a triage system for hospital services.^4^

To address concerns about misdiagnosis, mechanisms have been built into the online platform. Practitioners using the platform can only deal with 98 listed conditions. If a patient’s condition is not on the list, the platform’s algorithm suggests that the physician refer the patient to a hospital. All video consultations are recorded, allowing for quality control on randomly-selected patient encounters. Since its establishment in 2012, the platform has processed over 8 million consultations.

However, challenges exist. First, in China, primary health-care providers do not serve a gatekeeping role and patients can freely seek specialist care. Second, the mean age of village doctors, the main primary health-care providers in rural areas, is now 49.3 years, and retiring doctors are hard to replace.^5^ Village doctors lack training opportunities.^6^ Health facilities depend on the revenue generated by prescribing medicines to patients.^1^ Online consultations are currently not covered by health insurance plans. 

Many tertiary hospitals allow patients to easily bypass primary healthcare providers by making online appointments directly. Some form of gatekeeping within the digital health system is needed to maximize its efficiency. The platform could be used to empower primary health-care providers in the interaction between patients and the health-care system. The platform should also facilitate online communications between primary health-care providers and hospital physicians, allowing for more efficient triage than when patients self-refer. However, it is not yet clear whether this model can improve coordination of services, reduce pressure on secondary and tertiary hospitals and maximize efficiency.

Digital health services could help improve access for the rural population. Of the 14 000 connected providers, 55 are village doctors in Guangdong, who can now consult hospital physicians and let patients talk to physicians through the platform.^7^ The provincial government intends to roll out this platform to 2277 clinics in poor villages in Guangdong, with financial support of 30 million Chinese Yuan (about 4.5 million United States dollars). 

Physicians using the platform are less likely to overprescribe, with patients having to fill their prescriptions at local clinics or pharmacies, where the average cost of medicines is 75% of the cost per prescription in the provincial capital, Guangzhou. A potential consequence of fewer unnecessary prescriptions, however, is decreased income for community-based providers, which may disincentivize these providers from using the platform. Therefore, an innovative incentive structure may be needed to retain providers. 

Funding for the platform has come from government grants, the provincial hospital’s funds and a medical technology company, causing concerns about financial sustainability. In response, in June 2018, Guangdong province passed an action plan to ensure that in future, primary health care provided through digital platforms will be covered by insurance.^8^

Digital health in China faces similar challenges to those found in other countries, including patient safety, data security and a lack of oversight and evaluation frameworks.^9^ This platform in Guangdong may provide an example of how to improve the community-based primary health-care system in China.^10^ Policy-makers can use this example to incentivize and empower primary health-care providers, to improve the quality of care in remote and rural areas and to extend insurance coverage to primary healthcare provided through such a platform. 
